# Supporting Aging in Place: A Relational and Preventive Approach to Integrated Care in Naturally Occurring Retirement Communities

**DOI:** 10.5334/ijic.10601

**Published:** 2026-10-23

**Authors:** Shoshana Hahn-Goldberg, Jen Recknagel, Melissa Chang, Cecile Raymond, Kathrin Brunner, Miao-Ying Huang, Paulina Bleah, Ana Macpherson, Alicia Remark, Leslie Williams, Rachelle Patille, Sandra Yue, Howard Abrams

**Affiliations:** 1 NORC Innovation Centre, University Health Network, Toronto, Ontario, Canada; 2 Leslie Dan Faculty of Pharmacy, University of Toronto, Toronto, Ontario, Canada; 3 Department of Medicine, University of Toronto, Toronto, Ontario, Canada

**Keywords:** community-embedded, prevention, aging in place, naturally occurring retirement community, integrated care

## Abstract

**Introduction:**

Naturally Occurring Retirement Communities (NORCs) offer a unique opportunity to deliver integrated care within existing housing where older adults already live. In this context, NORCs are defined as high-rise buildings where at least 50 residents and at least 30% of residents are aged 65 and above. This paper presents an implementation case study of the NORC Innovation Centre’s (NIC) integrated care model (The UHN NORC Program).

**Description:**

The UHN NORC Program partners with housing, community, health, and social care providers to enable NORC buildings to adopt community-embedded care. An interprofessional care team collaborates with residents and local organizations to co-design building-level health and wellness programs. Across two Toronto NORCs, the program engaged 60% of residents, with 14% (n = 109) referred for individualized support. Relational care practices that emphasized trust-building, advocacy, and strength-based engagement were key enablers of success, along with the team composition and structure, system partnerships, and community leadership.

**Discussion:**

This model demonstrates how integrated care can be embedded within existing housing infrastructure using a relational, team-based approach that links health, social, and community systems.

## Introduction

The population of many countries is aging [1]. Additionally, older adults overwhelmingly want to age in place, with 96% of Canadians aged 65 and older reporting that they would do everything they could to avoid going into a long-term care facility [2]. However, many older adults require health and social services that cannot keep up with the demand [3]. Traditionally, Naturally Occurring Retirement Communities (NORCs) are defined as high-rise buildings where at least 50 residents and 30% or more of residents are aged 65 and above [4]. NORCs most commonly form in cities, where older adults either remain in these buildings as they age or move to them from single-family homes after life transitions [5]. Recent research in Ontario shows that older adults living in NORCs are generally older, more likely to be women, live alone, have lower incomes, and use the health system more frequently than non-NORC residents [6], suggesting that NORCs are important targets for equity-focused strategies such as community-led integrated care.

Community-led integrated care is increasingly recognized as essential for building equitable and effective health systems [7,8]. Due to the higher density of older adults residing at the same address, NORCs offer a unique opportunity to deliver integrated, community-based care within existing housing. Furthermore, a community-embedded integrated care approach shifts the focus from service delivery that is reactive to proactive, enabled through shared effort between communities and care providers.]. This is different than most integrated care programs, which focus on reducing future hospital utilization following a hospital visit [9,10].

This paper presents an implementation case study of a NORC-based integrated care model, supported by a descriptive mixed methods evaluation of implementation and early outcomes.

## Description

### Context

The NORC Innovation Centre (NIC) at University Health Network applies a community-embedded approach to integrated care through its work with older adults living in NORCs located in Toronto, a large urban city in Ontario, Canada. Toronto is one of the most multicultural cities in the world. Additionally, of the more than 425,000 older adults in Toronto, 20% fall in the low-income category. These disparities are further exacerbated for women and visible minorities [11]. This provides an unmatched opportunity to understand NORC Program delivery and produce transferable knowledge. There are currently 2,260 NORCs housing 268,166 older adults in Ontario, which is more than the number of older adults living in long-term care and retirement homes combined [12].

In addition to creating tools and resources to support the practice and policies needed for governments and other groups to implement integrated care programs within NORCs, the NIC has a living laboratory of 30 NORC buildings to test specific programming approaches and determine best practices. The 30 buildings were chosen in a way that prioritized high health and social needs, while requiring high levels of resident and building partner interest [13]. Within the living laboratory, the NIC has been delivering the UHN NORC Program model, a supportive integrated care program, at two NORC buildings. The program began in September 2023 and at the time of preparing this report, was in place for 2 years. Other buildings in the laboratory did not have onsite support or access to the integrated care team for individualized support, the focus of the case study described below.

The authors of this case study have first-hand knowledge of the program as front-line integrated care providers (MH, AM, AR, PB, HA, LW, RP), managers (KB, CR, SY), NIC leadership (HA, MC, JR) and evaluators (SHG).

### The program

#### Overview

The UHN NORC Program model is a community-embedded integrated care intervention that strengthens access to health and community care supports for older adults at the building level, while supporting a coordinated delivery system across the community, health, and housing sectors. The model applies relational care approaches that prioritize human connection, trust, and meaningful relationships between residents of NORC buildings, families, and care providers. The model has three mutually dependent components designed to engender trust, local adaptation, and system integration.

The first component is local leadership. Health systems often struggle to engage older adults meaningfully. Activating local leadership builds trust, ensures solutions reflect local needs, and aids in program sustainability. Within local leadership, key elements include local champions and peer-to-peer networks. Local champions (referred to as NORC Ambassadors) are resident leaders, trained through the NIC’s ‘NORC- Ambassadors’ curriculum. Local leaders amplify resident voices for meaningful change, foster a network of neighbours to provide peer-to-peer support by helping with everyday tasks and community initiatives, and create a sense of belonging and mutual care, while complementing formal services. Ambassadors also initiate social and recreational programming such as coffee mornings, lunch and learns, and various common interest groups.

The second component is onsite support. With large numbers of older adults in the same buildings, NORCs offer the opportunity for efficient, place-based services that are more accessible, easier to navigate, and relational. Located within NORC buildings, the UHN NORC Program model offers onsite support through community connectors (CCs), group programming, and property management support. A CC is a part-time onsite staff member who builds trust, fosters social connections, links residents to services, and coordinates programs. Group programming focuses on social interaction, prevention and health promotion, making care accessible and proactive. Property management support enables the use of dedicated community spaces, which support social interaction, program delivery, and service access, critical for building trust and engagement.

The third component is integrated health and social care. The program addresses common system challenges, such as fragmentation and lack of follow-through by ensuring clarity, continuity and agency for older adults. The model leverages existing formal health and social systems to work as one team. Key elements include an integrated care team and partner network. The integrated care team includes the CC described above and access to individualized support through an integrated care lead (ICL) and nurse practitioner (NP), especially for chronic or complex needs. Core functions adapt to regional structures and include navigation, care coordination, linkage to a resident’s usual circle of care, or primary care attachment and escalation to hospital or specialists. The NIC partner network includes over 65 health and social care partners that deliver programs such as exercise classes, wellness clinics, foot care, falls prevention and digital literacy in NORC buildings. Embedding services onsite reduces duplication and improves efficiency.

Altogether, the program is a novel way to bring integrated care into the community. The program takes a population health approach and is dedicated to older adults aging safely in community, addressing health and social needs together. The care team is embedded directly in NORC buildings, providing visible, ongoing support where older adults live, and uniquely connecting healthcare expertise with grassroots social support and community activation through the NORC Ambassadors. Additionally, the care team explicitly drives program evaluation and continuous innovation, using learning to shape iterative program development.

##### Involvement of People with Lived Experience

NORC Ambassadors are central to the model. They actively engage the community through regular touchpoints to understand the needs of residents and identify priorities. They are also integral to peer support networks that form as ways for neighbours to check in on each other and help when needed. They take the lead on organizing social and recreational programs that help foster community. In addition to the ongoing and integral role of NORC Ambassadors, several older adult community members were directly involved in the initial design of the integrated care program, and the community has been involved in providing ongoing feedback to support iterative improvements to the program.

#### Care Team Roles

The NORC Care Team works closely with residents and home and community care providers to bring ‘one team’ together to deliver the integrated care program. Each team member plays an important role in ensuring the success and efficiency of the program.

##### Community Connectors (CCs)

CCs are on-site support for the NORC Ambassadors and residents of the NORC. At the NORC Ambassador level, CCs support the formation and development of NORC Ambassador groups and networks of volunteers, coaching them to lead and sustain local initiatives. They apply critical thinking and conflict resolution skills to navigate any complex community dynamics. CCs work with NORC Ambassadors to identify their buildings’ collective needs, values, and goals to initiate programs that support aging in place, engaging partners as needed. CCs also support community-led initiatives that focus on common health conditions faced by older adults, as well as other needs, such as improving digital literacy, and social and community building activities in general, as identified by the ambassadors and the community.

CCs spend between two and three days on site in the NORC building. At the resident level, CCs connect through regular drop-in hours, and one-on-one as needed. CCs use relational care approaches to support residents in several ways, including:

- Providing personalized guidance and emotional support
- Advocacy support to ensure their voices are heard and respected
- Supporting health care navigation inquiries by providing residents with appropriate information and resources
- Connecting residents to health and social programs within and outside the NORC that do not require clinical or healthcare referrals
- Facilitating social connections, inclusion, and belonging among residents through purposeful and culturally sensitive engagement

CCs refer residents with more complex care needs to the ICL and NP for individualized support. In those cases, CCs contribute to the review and care planning, providing education and a mechanism for follow up.

Additionally, CCs establish relationships with new and existing service provider organizations, educating them on the UHN NORC Program model and the community-embedded and relational approach to community development. They build relationships, alongside the NORC Ambassador groups, with building managers, other site staff, and governance structures, such as boards of directors. CCs also keep track of innovative practices from NORC sites that can be scaled to other NORCs.

##### Integrated Care Leads (ICLs)

ICLs are registered health professionals from a variety of allied health professions (e.g. occupational therapists, respiratory therapists, pharmacists, etc.). ICLs provide a single point of contact to help with timely assessment and comprehensive care coordination, addressing system gaps and working across health, social, and community system partners to create one team of support. The ICL supports residents who require individualized health or social support, and their caregivers, as well as supporting residents through the development of clinical pathways.

ICLs may visit residents in their home, or have virtual or hybrid visits, where some members of the team are virtual and others are in-person with the resident. They develop a full appreciation for the complexity of a resident’s health situation, applying relational inquiry to explore concerns and possibilities for meaningful changes to support residents including:

- Developing individualized care plans, and providing health education and clinical guidance to support self-management
- Referring residents to relevant health-related service provider organizations such as those providing home care and community support services, using effective communication, information sharing, coaching, and follow through with residents and partners to ensure successful referrals
- Advocating for more advanced care at home, and avoiding unnecessary emergency department visits and hospital readmissions

Additionally, ICLs lead the development of clinical pathways and refine these pathways based on resident feedback. Through the pathways they develop, ICLs also deliver health education and promotion initiatives at the building level, such as falls prevention and COPD action plans, and coordinate these initiatives based on health trends, needs, and gaps.

ICLs bridge siloed services and disciplines through relational leadership, cultivating trust, empathy, and shared accountability amongst service providers. This involves motivating working together, creating enabling conditions, balancing multiple perspectives, and effectively working with power dynamics. They explore the impact of experiential changes to support meaningful and sustainable health changes, participating in social and recreation programs to deepen the view of ICLs as both care providers and program participants.

##### Nurse Practitioner (NP)

The NP further extends the scope of the integrated care program. Gaps in care often include a need to escalate care in the community to avoid unnecessary emergency department visits and many residents lack or lack timely access to a primary care provider (PCP) [14]. The NP is able to support a resident’s PCP were necessary, by ordering tests, referring residents to specialists, and mobilizing community paramedicine as needed.

In addition to working together with the ICL on understanding the complexity of a resident’s full situation and building customized plans, the NP can advocate for the resident and integrate their activities with the resident’s other care providers to create a seamless support system. The NP may add another layer of clinical support to both resident and PCP by:

- Completing comprehensive health histories and assessments
- Formulating diagnoses
- Prescribing medications
- Ordering and interpreting lab tests and diagnostic imaging.
- Enlisting home-visiting Community Paramedic services

For mutual residents/patients, the NP collaborates with PCPs. The NP can extend the reach of primary care, a necessary support given the current shortage of primary care across the globe [14]. If the resident does not have a PCP, the ICL will help connect them and the NP will act as a PCP for them until they secure an alternate. If the resident does have a PCP, the NP may still assume the role of most responsible provider in specific, well-defined scenarios such as urgent or time-sensitive care needs that arise, and more directly managing transitions of care such as post-hospital discharge, and advanced or palliative care planning. In these cases, the PCP continues to manage all other aspects of their patient’s care. This shared-care model allows for flexibility, responsiveness and continuity, ensuring that residents receive the right care, from the right provider, at the right time.

In certain cases, the NP also works closely with Community Paramedicine, a program where paramedics can deliver on-site wellness checks in the home and support care escalation. The NP provides focused assessments for residents in NORC clinical pathways, such as COPD lung health, and refers residents to health programs like diabetes education and pulmonary rehab, and to community care providers or specialists such as physiotherapists and dermatologists.

#### Implementation of Individualized Support

Although most NORC residents do not require specialized individualized services, the targeted support is important for the smaller group that may fall through the cracks or rely on inappropriate levels of the formal health sector.

##### Eligibility

To qualify for individual support from the ICL and NP, individuals must be at least 55 years of age and reside in a NORC building within the NIC’s learning laboratory. The program also extends support and guidance to family members of the eligible older resident. This includes adult children or dependents living in the same household who may have complex needs, such as cognitive, physical, or developmental challenges, often requiring informal caregiving from the older adult family member.

##### Referrals

The majority of referrals to the ICL and NP are made by the CC directly, and occasionally by community paramedics. However, ICLs occasionally attend health-related programs in the building, where they interact with residents directly and provide further clinical follow up if needed. Additionally, once connected, individuals can self-refer. Relational practice helps ensure residents are comfortable sharing their concerns with the NORC Care Team. Additionally, taking the time for a thorough assessment supports earlier identification and intervention, supporting prevention. Residents are followed by the NORC Care Team until their health concern is resolved or the resident is stable or connected with primary care for their ongoing needs.

##### Care Planning and Coordination

Once a resident is referred to the ICL or NP, collaborative care planning is conducted in partnership with the resident, ensuring their voice, values, and choices are central to all decisions. A standardized care planning template, developed by the NIC, is used to guide consistent and comprehensive documentation. A thorough assessment of the resident’s physical, emotional, social, and cognitive needs is conducted at the intake appointment and regularly reviewed. As part of the program, residents have a personal wellness plan that outlines their unique health goals, preferences, and strategies for achieving optimal well-being. The plan addresses multiple dimensions of wellness, including physical health, mental health, social engagement, spiritual needs, and lifestyle preferences. Residents are encouraged to take an active role in setting and reviewing their wellness goals, fostering autonomy and relational engagement. The team also makes use of standardized care pathways, which are available for common health conditions and care needs (e.g., dementia, diabetes, mobility support), ensuring consistency and quality across care teams. While standardized, these pathways are adapted to align with residents’ personal wellness plan and evolving needs.

##### System Coordination

The program uses a partnership approach, working with the broader network of health and social providers to support the needs of the building residents and individual cases referred to the ICL and NP. The breadth of partners and services spans traditional health, social, mobility, and any other service that the resident may need. The NORC Care Team serves as a central point of coordination, ensuring that all parties are aligned and that the resident’s expressed values remain supported throughout their journey. The team continues to engage with the resident at every step, helping them navigate the system and ensuring that transitions between services are smooth and complementary.

##### Supportive Organizational Practices

There are several organizational practices that support team formation and effective functioning across the NORC Care Team. Firstly, there are weekly sessions dedicated to promoting innovation, integration, and shared problem solving that all members attend along with management. Secondly, there are weekly clinical rounds led by the Care Team to support clinical decision making and team learning. There is a physician medical lead that attends the rounds and is able to weigh in on more complex situations. Additional touch points across the team focus on reviewing daily operations and aligning activities with strategic goals. Standardized procedures have been developed and used to ensure seamless handovers and to enhance safety and coordination during checking in and out from community-based work. Finally, there are regular education sessions to build capacity in key areas such as humanistic care approaches, equity diversity and inclusion, community partnership development, environmental awareness, self-protection and disengagement skills, dementia care, and reviewing learnings for the purpose of iterative program development.

### Evaluation methods

The evaluation of the integrated care program used an implementation and effectiveness design with two primary aims [15]. One was to understand the implementation of the program including facilitators and barriers, and the other was to assess its effectiveness in meeting the program objectives. The ultimate objective of the NIC’s integrated care program is to increase the ability for older adults to age in place in NORCs. To study implementation and impact, there were four primary sources of data analyzed (Table 1).

**Table 1:** Data sources for the evaluation of the UHN NORC Program model.


DATA SOURCE	DESCRIPTION	POPULATION	TIMING

Resident Wellness Survey	Survey, co-created with NORC residents, includes questions assessing health and wellness, participation, and feedback on the most significant changes experienced due to the program	Anonymous cross-sectional survey of all building residents	Done at baseline in Summer 2023 and again in Summer 2025

Chart Review	Detailed chart review of all visits and notes in residents’/patients’ electronic charts	Residents who receive individualized services from the ICL and/or NP	Chart review completed July 2025, after first two years of program

Program Tracking	Records of group programs occurring within NORC buildings including program type and attendance	Anonymous tracking collected at each building	Ongoing

Staff Reflections	Program staff working with NORC buildings and residents record regular reflections on factors impacting the programming. CCs track any time they interact with or support a resident.	Reflections entered by all staff including CCs, ICLs, and NPs	Ongoing


For the analysis, the NIC adapted the Community Health Guidance for Policy and Systems (Community Health GPS), a comprehensive framework designed to bridge local level action and system level learning [16]. The Community Health GPS includes four interconnected domains – place, people, public system, and policy-practice learning. These were adapted to represent the elements of the NIC integrated care program: (1) Healthy Communities – creating caring and connected communities; (2) Healthy People – supporting individuals to focus on their health and wellness; (3) Healthy System – improving appropriate access to services and information; and (4) Healthy Growth – using learning and continuous improvement to improve reach and sustainability. Key metrics were chosen within each domain (Table 2).

**Table 2:** Metrics by impact domain to study implementation and effectiveness of the NIC integrated care program.


METRIC	DESCRIPTION	RELEVANT DATA SOURCES

**Healthy Communities**		

Improvement in supportive community networks	Self-report of top two response options of agree or strongly agree (Likert scale) to having neighbours to call on for support *Question adapted from the Canadian Community Health Survey (CCHS)	Resident Wellness Survey

Improvement in sense of belonging in the building community	Self-report of top two response options of agree or strongly agree (Likert scale) to feelings of belonging in the building *Adapted from Question 10 of the Lubben Social Network Scale	Resident Wellness Survey

**Healthy People**		

Improvement in physical and mental health	Self-report of top three response options from good to excellent (on a five point scale from poor to excellent) physical and mental health *Question from the CCHS	Resident Wellness Survey

Improvement in comfort with technology	Self-report of the top two response options of intermediate or advanced (on a five point scale from none to advanced) level of comfort *Question regularly used by Tech TO	Resident Wellness Survey

Individual health and wellness needs addressed	Recording of issues addressed through individualized support from ICLs and NPs	Chart Review

**Healthy System**		

Health system navigation and connections	Referrals to health services including primary care, home care, and other community services	Chart Review

Improvement in inappropriate health system utilization	Reduction in avoidable emergency department visits	Chart Review

**Healthy Growth**		

Increased reach	Proportion of older adult residents participating in programming, interacting with the CC, and referred to individual support with the ICL and/or NP as identified by a yes/no checkbox on the survey or existence of a chart for review	Resident Wellness Survey and Chart Review

Factors impacting program implementation and sustainability	Qualitative data exploration	Staff Reflections


*CCHS 2023: https://www23.statcan.gc.ca/imdb/p3Instr.pl?Function=assembleInstr&a=1&&lang=en&Item_Id=1496615; Lubben Social Network Scale: https://www.brandeis.edu/roybal/docs/LSNS_website_PDF.pdf; TechTO unpublished survey: https://www.techto.org/.

All quantitative data was analyzed descriptively and when appropriate, comparing responses between baseline values and after two years. Since surveys use a cross-sectional anonymous sample, we cannot be sure that the same people completed both the pre and post surveys. Quantitative analyses were exploratory and not designed to test hypotheses or establish causal relationships. Qualitative data was analyzed using inductive coding. The evaluation was approved by the Quality Improvement Review Committee at the University Health Network and exempt from full Research Ethics Board Review (QIRC#2*3-0549*).

### Results

There are 777 older adults aged 65 and above across the two buildings, comprising 63% of the buildings’ residents. Average age is 78 and 60.2% are female. One building is a condominium and one is co-operative housing. Both buildings are in neighbourhoods with high diversity populations and lower income.

There were 186 survey responses at baseline and 173 after two years of the program. Demographics from the baseline and subsequent surveys were similar. Approximately 90% were from residents aged 65 and above, representing just over 20% of the older adult residents. Of those that responded, 70% were female, 11% were informal caregivers for loved ones, and 30% had a disability. The proportion of respondents living alone was 30% in the condominium and 70% in the co-operative building. While almost all respondents from the co-operative building speak and responded to the survey in English, 33% of respondents from the condominium speak languages other than English at home and 5% of survey responses were completed in Traditional Chinese.

At the two NORCs, during the first two years of the program, the ICL and/or NP saw 109 residents for individual support. Sixty-one residents (7.9% of the older adult residents) were seen in the first year, and 48 residents (another 6.2%) were seen in the second year. The average number of new residents referred each month was 4.4 (0 to 11), which has gone down over time, from 5.1 referrals per month in the first year to 3.7 in the second year. Between 15 and 20 residents are actively followed at a time. The average age of residents referred was 79.1 years old and 76.1% were women, with 32.1% living alone. Just over half (51.4%) have caregiver support, and 25.7% having public home care. Almost all (89.9%) have a primary care provider.

To understand the complexity of the residents, we pragmatically created a classification for program operations, using the following scale. This classification has not undergone formal psychometric validation:

1. Complexity level 1 (simple) is for a single straightforward request or receiving a falls pendant as the only activity.
2. Complexity level 2 (moderate) is for a resident requiring care navigation and referrals or patient education. Filling in for standard primary care is also in this category.
3. Complexity level 3 (complex) is for residents requiring significant navigation or if they have multiple requests with complicated issues.

Residents with the highest level of complexity were less likely to be female, but more likely to live alone, have an informal caregiver, and have both primary care and homecare (see Table 3).

**Table 3:** Demographics by complexity level.


	COMPLEXITY 1 (n = 65, 59%)	COMPLEXITY 2 (n = 34, 31%)	COMPLEXITY 3 (n = 10, 10%)

Average age	80.2	77.8	77.5

Percent female	75%	71%	50%

Percent living alone	32%	38%	50%

Percent with an identified informal caregiver	47%	50%	80%

Percent with a primary care provider	87%	93%	90%

Percent receiving government funded homecare	25%	24%	50%


#### Healthy Communities

The most significant change noted in the qualitative responses on the survey was an increase in social interaction and sense of community. People highlighted getting to know their neighbours and growing to appreciate them and their cultures. People remarked that it is nice to belong to a supportive community and that the increase in the closeness of the community is noticeable. This was explained to be especially valuable to those who live alone. Additionally, people mentioned how staying engaged helps improve loneliness and mental health. Those who volunteer mentioned that they enjoy it, bringing them joy.

On the survey, those reporting that they agree or strongly agree to having a sense of belonging in their building was high at baseline at 92% and increased to 94% following two years of the program. Those reporting that they agree or strongly agree to having neighbours that they can call on for help increased from 80% at baseline to 91% following two years of the program.

#### Healthy People

On the qualitative survey responses people highlighted that they noticed a significant change in how they take care of themselves, and that having a caring and connected community leads them to participate in more programming. People reported that the ability to exercise and get their health checked in their building was helpful and that they are more involved, eat better, exercise and socialize more. Specifically, having more exercise programs in the building was mentioned as being helpful.

On the survey, those reporting good to excellent physical health remained constant at baseline and following two years of the program at 69%. Those reporting good to excellent mental health increased from 82% at baseline to 88% following two years of the program. Those reporting having an intermediate or higher level of comfort with technology increased from 44% at baseline to 63% following two years of the program.

For the 109 residents seen for individualized support, there were 146 separate referrals over the two-year period, with 30.3% of residents having more than one referral. There were an average of 35.5 days and a median of 13.5 days from referral to resolution, showing that the majority of referrals are more straightforward, with a smaller percentage being more complex or taking a longer time. The minimum referral length was 1 day, and the maximum was 335 days. On average, each referral has 3.2 encounters (median = 2) before it is resolved. Table 4 provides reasons for referrals, as well as reasons for re-referrals and additional issues addressed through individual support. Falls prevention was the most common, followed by home accessibility and mobility needs and homecare support.

**Table 4:** Top reasons for and issues addressed during referrals.


REASON/ISSUE ADDRESSED	ORIGINAL REFERRAL REASON	RE-REFERRAL REASON	ADDITIONAL ISSUE ADDRESSED	TOTAL

Falls prevention	56	8	7	71

Home accessibility modifications, mobility assessment and mobility equipment needs	4	6	11	21

Illness or injury	5	9	2	16

WheelTrans application or other disability paperwork	4	8	2	14

COPD screening	5	6	0	11

Chronic disease management support	3	1	6	10

Primary care provider attachment needs	8	1	0	9

Patient education	1	5	2	8

Personal support worker and other homecare needs	3	2	2	7

Medication management support	3	2	2	7

Mental health concerns	1	2	3	6

Caregiver respite or support	5	1	0	6

Pain	2	3	1	6

Post-hospital discharge support	1	4	1	6

Medical devices (e.g. hearing aids, CPAP)	3	1	1	5

Dementia support	3	0	1	4

Sleep issues	2	2	0	4

Grief counselling	2	1	0	3

Goals of care discussions	0	0	2	2

Housing issues and planning	1	0	1	2

Meals on wheels or other nutrition issues	0	1	1	2


#### Healthy System

On the qualitative responses survey, several people mentioned that the most significant change they experienced due to the integrated care program was an improvement in comfort and feelings of safety because they have a CC in their building and they know they can go to them with questions or for help. They mentioned that it was valuable to have someone dedicated to addressing their concerns and enhancing their relationships. People mentioned that they feel more confident in their ability to age in place because of the NORC Program and know that if health issues arise, they will be more likely to get the support they need. People expressed gratitude for having in-building access to so many services at no or low cost.

The integrated care program, and the individual support in particular, aims to fill system gaps and act as a bridge supporting and connecting residents as they navigate their care needs and ensures residents receive appropriate care. For the 109 residents who received individual support, 152 connections were made to health and social services (see Table 5). The two most common services were the remote care monitoring falls pendant program, with 51 (46.8% of people) referrals, and homecare, with 29 (26.6%) referrals.

**Table 5:** Connections made by the integrated care team.


CONNECTION MADE	COUNT

Falls pendant remote care monitoring program	51

Homecare – personal support worker (12), physiotherapy (8), nursing (1), housekeeping (2), home safety and accessibility assessments (6)	29

Outpatient clinics – spirometry, foot care, pain, physiotherapy, naturopathy, sleep, limb preservation, hypertension, geriatric psychiatry	18

Transportation programs	10

Mobility devices	10

Specialists – endocrinology, ophthalmology, respirology, dermatology, rheumatology, physiatry, nephrology, palliative care	9

Primary care	7

Community paramedics home visits	4

Nutrition (meals on wheels and dieticians)	3

Grief counselling (Care First and Catholic Family Services)	2

Imaging (ultrasound and CT scan)	2

Pharmacy (e.g. med-rec)	2

Rehabilitation (cardiac, inpatient)	2

Supportive housing	2

Caregiver respite (providence overnight respite and OCO)	2

**Total connections**	**152**


Another gap filled is post-hospital discharge support. In five specific cases, residents were supported by the integrated care team post-hospital discharge in a way that enabled them to return home sooner with supports instead of requiring an alternate level of care or remaining in hospital, with an alternate level of care designation. There have also been nine documented cases of ED visits that were avoided, as outlined in Table 6.

**Table 6:** Methods of ED avoidance.


METHOD OF AVOIDANCE	COUNT

Reassurance about symptoms	2

Post-fall arranged community paramedic visit and/or outpatient CT scan	2

Community paramedic home visit about urgent but not emergent symptoms (e.g. seizure)	4

As part of a plan to call and monitor symptoms as part of GOC while palliative	1

**Total**	**9**


#### Healthy Growth

Over the two-year period, the program reached 466 older adult residents (60%) across the two buildings, who were supported by the CC or participated in group programming, and including the 109 who received individualized support.

The analysis of staff reflections resulted in four significant program facilitators: (1) team structure and composition, (2) having a relational approach, (3) system integration, and (4) community leadership.

##### Team Structure and Composition

The hybrid nature of the team, where the CC is on site at the NORC building facilitates care plan implementation. The CC checks in with residents and provides education and advice in between ICL and NP visits. Participation in some in-building activities can also be included as elements of a resident’s care plan. Additionally, early relationship development between the CC and residents develops trust that extends to the ICL and NP. This relationship is reciprocal, with the CC valuing being able to consult with the ICL and NP easily. It is also important that all members of the care team are a neutral third party, rather than being employees of the NORC building, especially when social factors are present. For example, the team has been able to support residents facing housing or eviction issues while also being a support to the housing partner. This can also be valuable when there is management change or turnover in building staff.

##### Having a Relational Approach

A unique feature of the program is the emphasis by the team on relational care approaches, which have been extraordinarily valuable at creating trust. The team is trusted even when there is fear of many others and in cases where there is previous trauma or poor experiences in the health system. The NORC Care Team establishes trust through relational presence by seeing the person as a whole, holding non-judgmental space, and acknowledging the person’s inherent strengths, wisdom, and expertise. In a small number of cases, intense accompaniment has been required to support trust issues and ensure successful transitions to referred services. For example, one resident had been labelled ‘non-compliant’ because they did not trust a specific care provider due to past experiences. Through relational inquiry, the NORC Care Team listened deeply to the persons lived experience, using curiosity to truly understand the persons needs and challenges, supporting the persons expressed care values to be honored for a successful transition to needed care. Additionally, accompaniment and education also supported the resident with a successful transition to needed care. The resident expressed being “no longer alone in a dark tunnel.” Mistrust in the healthcare system has been identified as a significantly negative predictor of trust with providers [17], highlighting the importance of using relational approaches to create an interconnected network of support that includes neighbours, caregivers, the CC, and the ICL and NP.

##### System Integration

The UHN NORC Program works collaboratively across health, housing, and community to build integrated support that meets people where they are, aligns with local priorities, and reduces duplication. The staff reflections and chart notes show that significant effort is put towards building strong partnerships and relationships with community organizations. These partnerships have proven valuable for the integrated care model and partners, who have stated that they have improved their reach and comfort with working with older adults. These partnerships have enabled smooth transitions and referrals, as well as services, supports and in-building programs, including health education workshops, clinics, and fitness programming. CCs can work with partners to adapt existing programming to meet the needs of each NORC building. An example of a critical partnership is the partnership with the community paramedics, who run in-building group wellness clinics and can support individual requests. There were four examples of the combination of integrated care team and community paramedic home visits working together, resulting in an avoided ED visit.

##### Community Leadership

The UHN NORC Program leverages local strengths and networks to build sustainable systems rooted in community needs and community expertise. In each NORC building, NORC Ambassadors lead programming in the building, taking on key leadership roles in setting up and supporting events and programs. For example, Ambassadors identify resident priorities, recruit volunteers, welcome new residents, promote programs, support outreach, and liaise with building governance. They also strengthen community supports and foster a sense of belonging. In the surveys, many residents expressed gratitude for the ambassador’s volunteerism and leadership. Finally, the ambassadors extend the program reach and sustainability. They have a pulse on their building community, are well-connected, and understand successes and pain points. Partners state that NORC Ambassadors on the ground help promote programs and drive more fulsome participation.

##### Challenges

There were also several challenges identified through the analysis. A key challenge in implementing the UHN NORC Program model was ensuring clear roles and responsibilities for the members of the care team, compared to other building staff, homecare staff, and primary care. Without clear roles and responsibilities, the program risks confusing other providers and residents. Over time, and through the quality improvement mechanism in place, clear language explaining roles has been developed and embedded into orientation sessions. Secondly, given the uniqueness of the program, understanding and developing the needed skills and training for the care team was another challenge. Throughout the program implementation, these needs have been documented and the training program developed by the NIC has been continuously refined. A third challenge was adapting to the inconsistent use of technology by the older adult residents. The hybrid nature of the program has allowed flexibility to support residents at whatever their level of comfort with technology. Finally, significant effort was needed to ensure that relationships with partner organizations were strong and that the values of the UHN NORC Program model were incorporated into the role of partner organizations.

### Limitations

There are several limitations to the evaluation of the model. Specifically, surveys were cross-sectional and anonymous and there was no control group included. For these reasons, findings should be viewed as potential benefits and areas for future research as opposed to proof of causation.

## Discussion

Overall, the UHN NORC Program model was successfully implemented in two NORC buildings in Toronto. Unique elements of the program include the integration with the housing sector, leadership of older adult NORC residents, a focus on prevention by supporting older adults where they live, and the relational approach. The leadership of Ambassadors and inclusion of opportunities for strengthening community was likely instrumental in the noted changes people mentioned related to feelings of belonging and having the support of neighbours. Maintaining social connectedness throughout life is linked to higher rates of physical activity, mental well-being and improved cognitive functioning [18]. Additionally, the UHN NORC Program model worked collaboratively and enhanced the impact and reach of other community-based integrated care programs, such as the community paramedic program, which has highlighted the need to be nimble and experimental to meet evolving needs of community, and to identify unmet needs before emergencies occur [19].

The individual support component of the program functions as an idealized network, where any entry point allows access to a holistic care service. This is evident by the number of additional issues addressed outside of the initial or subsequent referral reasons. Once connected, the service acts as a gateway to address other issues. Key issues identified included falls prevention, mobility support, homecare coordination, and early intervention. Falls are the leading cause of injury in older adults and are a major cause of unwanted long-term care placement [20]. However, existing falls prevention programs may be inaccessible to the ones who need it most due to challenges with transportation, cost, or unequal service availability in disadvantaged communities [21,22]. Providing integrated and preventative care for falls prevention, co-designed with residents within NORC buildings, increases accessibility and appropriateness, and supports system efficiency, while having the added benefit of social connections that come from participating in community-based programming. Custom care pathways that include group components are part of the UHN NORC Program model and have already been implemented for falls prevention, while others will be developed for other identified common needs.

This focus on prevention extends beyond the individual support components to the broader NORC building community. For example, in-building exercise classes are a low barrier opportunity for older adult NORC residents to improve their joint mobility and movement, thus potentially avoiding chronic pain and future falls, which in turn can result in less healthcare utilization [23,24]. Interestingly, in some low-income countries, integrated care programs are more community based, with a stronger focus on prevention [25]. This is in line with recommendations that build on the World Health Organization framework, which supports integrating community determinants, service delivery, community organizations, partnerships, governance, and accountability, and recognizes communities as producers of health and agents of resilience [26]. Sacks et al. also note that community embedded models are needed for equity, to reach those who are the most isolated or who do not have access to primary care.

The immediate plans for the UHN NORC Program model include spread and scale. In September 2024, the model was spread to another NORC building, but at the time of this analysis, the model implementation was still in progress. Future plans include a supported adoption of the UHN NORC Program model by other agencies across Ontario, which will provide further insights into implementation of the model.
